# The interaction between dietary fiber and gut microbiota, and its effect on pig intestinal health

**DOI:** 10.3389/fimmu.2023.1095740

**Published:** 2023-02-14

**Authors:** Ruiqi Hu, Shuwei Li, Hui Diao, Chongbo Huang, Jiayou Yan, Xiaolan Wei, Mengjia Zhou, Peng He, Tianwei Wang, Hongsen Fu, Chengbo Zhong, Chi Mao, Yongsheng Wang, Shengyao Kuang, Wenjie Tang

**Affiliations:** ^1^ Livestock and Poultry Biological Products Key Laboratory of Sichuan Province, Sichuan Animtech Feed Co., Ltd, Chengdu, Sichuan, China; ^2^ Animal Breeding and Genetics Key Laboratory of Sichuan Province, Sichuan Animal Science Academy, Chengdu, Sichuan, China

**Keywords:** gut microbiota, dietary fiber (DF), microbial fermentation, short-chain fatty acids (SCFAs), intestinal health

## Abstract

Intestinal health is closely associated with overall animal health and performance and, consequently, influences the production efficiency and profit in feed and animal production systems. The gastrointestinal tract (GIT) is the main site of the nutrient digestive process and the largest immune organ in the host, and the gut microbiota colonizing the GIT plays a key role in maintaining intestinal health. Dietary fiber (DF) is a key factor in maintaining normal intestinal function. The biological functioning of DF is mainly achieved by microbial fermentation, which occurs mainly in the distal small and large intestine. Short-chain fatty acids (SCFAs), the main class of microbial fermentation metabolites, are the main energy supply for intestinal cells. SCFAs help to maintain normal intestinal function, induce immunomodulatory effects to prevent inflammation and microbial infection, and are vital for the maintenance of homeostasis. Moreover, because of its distinct characteristics (e.g. solubility), DF is able to alter the composition of the gut microbiota. Therefore, understanding the role that DF plays in modulating gut microbiota, and how it influences intestinal health, is essential. This review gives an overview of DF and its microbial fermentation process, and investigates the effect of DF on the alteration of gut microbiota composition in pigs. The effects of interaction between DF and the gut microbiota, particularly as they relate to SCFA production, on intestinal health are also illustrated.

## 1 Introduction

The gastrointestinal tracts (GITs) of mammals are home to abundant microorganism communities. As the largest interface between internal and external environments, the GIT is the habitat of the greatest number and diversity of microorganisms. It has been estimated that pig gut contains approximately 110 species of microorganisms, across 40 families and nine phyla (1). These microorganisms, including their genomes and extrachromosomal elements, interact with the host environment and are defined as gut microbiota (2–5). The ecosystem of the gut microbiota is complex and dynamic, and is involved in a symbiotic relationship with the host environment (6). Moreover, it plays a critical role in maintaining a healthy gut environment, further affecting nutrient utilization and physiological and immune function in the pig intestine (7, 8).

The gut microbiota composition of pigs varies and depends on GIT segment and pig age, sex, and diet, etc. It was reported that, in pigs from 11 to 12 weeks of age, the microbiota in the ileum was dominated by members of Bacillota, accounting for 90% of bacteria. In the cecum and colon, the proportion of Bacteroidota started to grow and accounted for approximately 40% to 60% of bacteria (9, 10). Apart from influencing the internal function of pigs, diet is also able to influence gut microbiota composition during the nutrient utilization process. Within the diet, dietary fiber (DF) supplementation plays a key role in influencing the composition of the gut microbiota, depending on its type, origins, and physicochemical properties, mainly because it escapes the digestive process in the small intestine and becomes available for microbial fermentation when it enters the distal ileum and colon (11, 12).

Although DF is not efficiently digested by enzymes, it is an integral part of the pig diet because it is a source of energy and has beneficial effects on intestinal health. The products of microbial fermentation of DF, short-chain fatty acids (SCFAs), are the main energy source of intestinal cells and maintain intestinal health and immune function (13). The benefits of DF and its fermentative metabolites on intestinal health drive new insights in the search for alternative strategies to antibiotic growth promoters (AGPs), which was initiated because of the ban on antibiotics issued in animal and feed systems worldwide (14). The objective of this review is to discuss the impact of DF on pig gut microbiota alteration, and of SCFA-mediated regulation on intestinal functioning and immunity. The conclusion will emphasize the importance of the interactive effects between DF and gut microbiota in influencing intestinal health and host health and performance.

## 2 Dietary fiber

A comprehensive understanding of DF has been developed thus far, with extensive studies conducted mostly in relation to the effects of DF on nutrient digestion, physiological and immune function, and intestinal health, depending on various DF characteristics (13, 15, 16). DF can be defined in various ways. Generally, DF represents the sum of carbohydrates that are undigestible by endogenous enzymes, namely non-starch polysaccharides (NSPs) and lignin (13, 16). These carbohydrates are mainly naturally present in plant cell wall components, including cellulose, hemicellulose, and pectin. Some non-cell wall components, such as resistant starch and some non-digestible oligosaccharides, possess effects comparable to those of NSP and lignin, and can therefore also be categorized as DFs (16). Common feedstuffs rich in fiber content include oats, wheat, barley, and by-products such as cereal hulls and distiller’s dried grains with solubles (DDGS) (13). The composition and physicochemical properties of DF vary widely in different feedstuffs and, consequently, have distinct functions in nutrient digestive processes. The composition of DF in different feedstuffs/crops is listed in **Table 1**. The major concern about DF, associated with its role in nutrient digestive processes, is related to its low energy value and negative effects on nutrient digestibility, which can also vary by DF characteristics. Despite its adverse effects on nutrient utilization, however, DF should be included in the diet at a minimum level to maintain normal physiological function and intestinal health. This section will provide a general introduction to DF to better understand the interaction between DF and the gut microbiota.

**Table 1:** Composition of DF commonly used in feedstuffs/crops*.

Item		MB	OH	RH	WB	PH	SB	RSH	SBP
*Chemical composition*	Unit								
DM	% as fed	88.70	90.30	91.90	87.00	91.60	89.10	87.50	24.30
CP	% DM	11.90	5.20	3.70	17.30	7.00	13.10	16.10	8.70
CF	% DM	12.30	30.60	42.60	10.40	65.90	38.90	27.30	20.80
NDF	% DM	44.20	75.80	67.80	45.20	66.40	64.40	55.80	49.50
ADF	% DM	14.50	36.00	51.70	13.40	56.40	46.20	42.20	24.80
Lignin	% DM	2.20	7.10	14.20	3.80	22.40	2.30	22.70	1.80
EE	% DM	4.60	2.20	1.50	3.90	2.00	2.20	13.20	0.50
Ash	% DM	5.80	4.60	17.50	5.60	5.20	5.20	5.50	6.80
Starch	% DM	35.00	9.90	5.30	23.10	–	5.20	6.00	0.50
Total sugars	% DM	2.80	1.20	–	7.20	–	1.60	2.60	5.20
Gross energy	MJ/kg DM	18.50	18.40	16.30	18.90	19.80	18.20	21.20	17.10

^*^Data were derived from Feedipedia. All the values represent the average or predicted value.

ADF, acid detergent fiber; CF, crude fiber; C, crude protein; DM, dry matter; EE, ether extract; MB, maize bran; NDF, neutral detergent fiber; OH, oat hull; PH, pea hull; RSH, rapeseed hull; SB, soybean hull; SBP, sugar beet pulp; WB, wheat bran.

### 2.1 Classification of dietary fibers

DF can be classified according to its constituents, type of oligosaccharides/polysaccharides, physicochemical properties, and physiological role in digestion. However, these classification methods do not completely cover all fiber categories (17), and, generally, the most accepted classification of DF is based on its solubility and fermentability. Fibers are classified into two categories in terms of solubility: soluble and insoluble fibers. The chemical structure of DF, and its interaction with water molecules, determines its degree of solubility. The insoluble fraction includes cellulose, part of hemi-cellulose, and lignin, forming a linear and ordered crystalline structure in the solution. Fiber sources containing a large insoluble fraction commonly utilized in swine diets include wheat bran, soybean hull, oat hulls, and DDGS, which are mainly plant co-products. The structure of soluble fractions, i.e., pectin, gum, and β-glucan, is highly branched, contributing to the increased solubility of DF (18). DFs with different degrees of solubility have different impacts on nutrient digestive processes and microbial fermentation metabolism (12, 15). It has been reported that soluble fibers are, generally, fermentable, whereas insoluble fibers are hardly fermented. Some soluble fibers are viscous, such as pectin, galactomannan, β-glucan, and psyllium, and others, including fructooligosaccharides (FOSs) and inulin, are non-viscous. Owing to their insolubility in water, insoluble fibers do not form gels and have little association with viscosity (19, 20).

### 2.2 Physicochemical properties

DF has different impacts on gut physiological function, largely associated with its physicochemical properties, i.e., solubility, viscosity, and water-holding/bonding properties (21). It has been found that insoluble NSPs (e.g., wheat bran) increased the average daily feed intake (ADFI) of weaned piglets by decreasing the mean retention time (MRT) of digesta along the GIT, whereas soluble NSPs (i.e., pectin and sugar beet pulp) tended to prolong the digesta MRT and increase satiety, consequently reducing the piglets’ feed intake (22, 23). Fermentation of soluble fibers starts in the ileum, whereas insoluble fibers are hardly fermented until entering the hindgut. Compared with insoluble fibers, soluble fibers are more easily degraded by microbial enzymes, contributing to higher levels of fermentation (24). Karr-Lilienthal et al. found that wheat bran containing a large insoluble fiber fraction resulted in poor fiber fermentation compared with sugar beet pulp with a high pectin content and soybean hull containing a high soluble fraction (25, 26). Moreover, soluble fibers can increase the viscosity of digesta in the small intestine (18). Thus, the viscosities of pectin and β-glucan are generally higher than that of cellulose in pig diets (27). Viscous fiber can bind water, leading to increased viscosity and modified digesta transit time. Thus, viscosity is an important factor affecting nutrient digestibility (28, 29). In addition, viscosity is likely to influence microbial fermentation by affecting colonic cells, a key source of energy, although it is not a dominant contributor to energy absorption (13). Fibers with low and high viscosities contributed to slow and rapid fermentation and SCFA production, respectively, by variably affecting digesta transit (30). Furthermore, DF combines with water to form a colloidal suspension, which is known as the water-holding capacity (31). Water-holding capacity, to some extent, determines swelling, that is, the solubilization and dispersion by the surrounding water of the DF structure (32). It has been shown that high fermentability is associated with high solubility, swelling, and water-holding capacity (33). Moreover, DF expansion and dispersion lead to easier access to microbial enzymes and promote fiber fermentation and SCFA production (34). In conclusion, it is critical to understand the physicochemical properties of DF, as they shed light on the mechanisms of DF that affect the physiological function of pig intestines.

### 2.3 Alterative to AGPs

DFs have been regarded as a potential alternative to antibiotic growth promoters (AGPs) since the use of AGPs has been banned or restricted in several countries (13). AGPs are efficient tools that increase the efficiency of transforming feed into animal products, and improve animal health and performance (35). However, the problem of increased resistance to bacteria of animal origin has been a great concern for human health throughout the world (35). Shang et al. have reported that DF can reduce diarrhea in postweaning pigs and improve their intestinal health by modulating the gut microbiota (36). Moreover, the metabolites of DF fermentation, especially butyrate, have been shown to benefit mucosa growth and increase water reabsorption in the large intestine by stimulating sodium absorption (37). Thus, rapidly fermented fibers, e.g., sugar beet pulp, might exert an anti-diarrheal effect (38). In addition, the use of DFs in the place of AGPs largely mitigates concerns related to the economic costs of producing the latter, particularly when antibiotics are also required for therapeutic or health-promoting purposes. Plant-derived compounds, such as tannins, are playing a cost-effective role in animal nutrition and leading to the development of a more demanding market (39). Overall, DF can be an effective alternative to AGPs because it positively modulates the gut environment and promotes the growth of beneficial bacteria, consequently improves pig health.

## 3 DF fermentation

Unlike ruminants, in which extensive fermentation occurs in the rumen, in monogastric animals, nearly all DFs escape the digestive process in the stomach and small intestine and pass into the colon, which is the major site of fermentation (13, 40). Studies have also found that substantial fermentation occurs in the distal ileum, where certain species of bacteria reside (14). Before fermentation starts, polysaccharides are broken down into smaller forms, or into monosaccharides, by microbial hydrolytic enzymes in a depolymerization process (13, 41). The rate of depolymerization largely determines how quickly carbohydrates become available for microbial fermentation (13). In addition, the degree of fermentability is associated with the physicochemical properties of the DF, that is, its solubility, water-holding capacity, and viscosity (41). Highly branched DF has been shown to have a larger surface area, which makes it more readily digestible by microbial enzymes, and, therefore, is more rapidly fermented (42).

**Figure 1:**
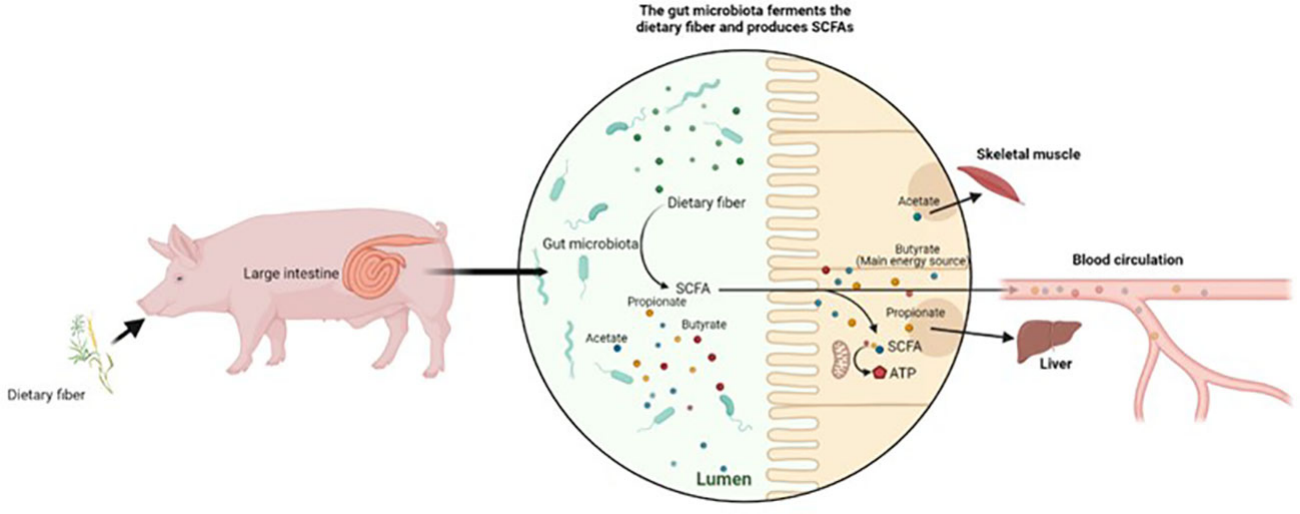
Microbial fermentation process and SCFA production.

The end products of DF fermentation (i.e., SCFAs) are considered the main energy sources of intestinal cells, and promote immune function and maintain intestinal health. The major site of SCFA absorption is the large intestine, where approximately 90% of SCFAs are metabolized (41). Acetates, propionate, and butyrate are the SCFAs most discussed when investigating microbial fermentation metabolites. The production of these major fermentation metabolites through microbial fermentation is illustrated in **Figure 1**. Acetate is the most abundant SCFA, accounting for approximately 90% of total SCFAs (13). Butyrate plays key roles in the proliferation of mucosal epithelial cells, and in strengthening the intestinal barrier, and is regarded as the main energy source for colonic cells (43). Butyrate can be synthesized by acetate and lactate when utilized by specific bacteria (44). Furthermore, the concentration of SCFAs varies along the GIT, with a lower SCFA concentration present from the cecum to the distal colon (45). A low amount of propionate was reported because most propionate is metabolized in the liver (46). Acetate was reported to be the most abundant SCFA in peripheral circulation, and to mediate the glucose metabolism and fatty acids utilization in skeletal muscle (47). The fiber fermentation process, and SCFA metabolism pathways, are reported and illustrated well in the study by Jha and Berrocoso (13).

Various studies have revealed that distinct DF characteristics can affect SCFA outcomes. A higher soluble fraction leads to an increased SCFA concentration in the small intestine, as soluble fibers are easily fermentable. Insoluble fibers, however, are fermented in the more distal parts of the GIT (48). Bai et al. observed that a higher concentration of acetate was produced by the microbial fermentation of xylan and xylooligosaccharide, whereas propionate and butyrate were produced in higher concentrations by the microbial fermentation of β-glucan and inulin (49); Ellner et al. conducted a study to compare the production of SCFAs when pigs were fed rye and rapeseed meal, and when they were fed wheat and soybean meal. The results showed that rye and rapeseed meal led to higher concentrations of SCFAs in the pigs’ jejunums and colons (50).

In conclusion, through DF fermentation, SCFAs are released as an energy source and help to maintain intestinal health. Fermentability and SCFA production largely depend on DF physical structures and chemical properties. DF fermentation enables the interaction between DFs and gut microbiota, which will be reviewed in the next section.

## 4 DF-microbiota interaction and intestinal health

### 4.1 Gut microbiota in pigs

In monogastric animals, three phyla, Bacillota, Bacteroidota, and Pseudomonadota, generally accounting for over 1,000 species of bacteria, predominate (13, 51). The species of bacteria colonizing the GIT vary in different gut segments. Zhao et al. analyzed the microbial population in four gut segments of matured pigs. They found that the predominant genera in the small intestine were aerobes, or facultative bacteria, whereas in the large intestine, the majority of the bacterial population were anaerobes. Pseudomonadota and Bacillota were the predominant phyla in the jejunum and ileum, and accounted for 70% and 20% of the total population, respectively. In turn, in the cecum and colon, Bacillota was the predominant phylum (> 75%), and Pseudomonadota accounted for approximately 13% of the total population (52). In general, the predominant bacteria along the GIT are *Streptococcus*, *Eubacterium, Lactobacillus, Clostridium*, and *Propionibacterium* (10). The gut microbiota profile in pigs is related to multiple factors, including their age, breed, health status, and diet. For newborn piglets, microbiota colonization mainly depends on their exposure to bacteria, including the sow and the gut environment. *Escherichia coli and Streptococcus* spp. are the initial colonizers, creating an anaerobic environment that favors the growth of *Bacteroides*, *Bifidobacterium*, *Clostridium*, and *Lactobacillus. Lactobacillus* dominates the microbiota profile because of its beneficial effect in inhibiting colonization by pathogens (53, 54). There is a dramatic alteration in the gut microbiota composition of weaning piglets when a new cereal-based diet is introduced, and this leads to the gut microbiota profile can becoming more specific, as it has been shown that *Prevotella* is more abundant and has a higher growth rate (55).

Fibers are broken down by microbiota and fermented into SCFAs for host utilization. DF acts as a substrate in the fermentation process and contributes to selective microbiota proliferation, resulting in the alteration of gut microbiota composition. The section below gives an in-depth overview of the alteration of microbiota composition affected by DF in terms of related studies in recent years. Furthermore, the factors involved in the DF–microbiota interaction will be discussed.

### 4.2 Effect of DF on microbiota composition

During the continuous digestion process of nutrients, a decreased amount of digesta flow enters the distal part of the GIT, leading to the alteration of fermentation metabolites and microbiota composition (56). Bacteria colonizing different GIT segments have been shown to have spatial heterogeneities that exert distinct effects on host health. It should be noted that the benefits derived from colonized bacteria are a result of the contributions of the whole microbiota community, rather than the effect of a single species (13). Spatial heterogeneity can be ascribed to different nutrient supplies for microbiota colonization in different segments (30, 57). Various studies have reported that the alteration of gut microbiota can be largely attributed to different DF characteristics. Generally, DF promotes the growth of bacteria species that are more capable of fermenting fibers than other species. In addition, a probable mechanism by which fibers can alter gut microbiota composition is that DF causes retained digesta; thus, more time is available for the proliferation of selective microbiota (13).

Wu et al. observed that Bacteroidota and *Turicibacter* were more abundant with increased crude fiber content in both the cecum and jejunum of growing pigs when soybean was the main fiber source (58); Ellner et al. also found a higher abundance of Bacteroidota in the colons of growing pigs fed with rapeseed meal (RSM) than in those fed with soybean meal (SBM) (50). This might be explained by the greater insolubility of an RSM-based diet, contributing to the growth of Bacteroidota, which in turn has been shown to increase with increased contents of resistant starch and maize bran, both of which contain large insoluble fractions (59). Moreover, there is evidence that a higher abundance of Bacteroidota is associated with weight loss (59, 60). In the study of Ellner et al., pigs fed an RSM-based diet were observed with reduced weight gain and higher abundance of Bacteroidota (58). Conversely, Luo et al. found a lower abundance of Bacteroidota in the colons of weaning pigs with increased galactose in the diet content when using pectin as the main fiber source. This might be explained by the high viscosity of pectin, leading to damage to the mucosal surface, thus modulating colonic morphology and bacterial colonization (61).

Heinritz et al. observed a higher abundance of *Lactobacillus* in the hindguts of growing pigs fed on diets containing a high content of NDF, which was similar to the results of Chen, Loo, and Heinritz (62–64), whereas it was observed that a lower abundance of lactobacilli in the ileum was associated with increased galactose (65). Chen et al. found higher abundances of *Lactobacillus* and bifidobacteria in the ileum and colon, respectively, with increased NDF content, in pigs fed wheat bran and pea fiber diets than in those fed a soybean fiber diet. Lower *E. coli* abundance was also observed in the ileum of pigs that were fed the wheat bran diet than in those fed the soybean fiber diet. The results showed that increasing DF modulates the gut microbiota, possibly in a pattern of promoting the growth of beneficial bacteria (i.e., *Lactobacillus* and bifidobacteria*)*, and suppresses the growth of pathogenic bacteria (i.e., *E. coli*) (66).

Thus, those fiber-degrading species associated with the particular physicochemical properties of targeting fibers can also affect bacterial colonization. For instance, actinobacteria and Bacteroidota are both common insoluble DF-degrading species, and their presence affects the performance and health of their hosts. It has been shown that an increased ratio of Bacillota-to-Bacteroidota reduces the incidence of diarrhea and infections (57). The results of recent studies related to the effect of different fibers on the alteration of gut microbiota composition of pigs at different growth stages are shown in **Table 2**.

**Table 2:** Results of studies investigating the effect of fiber on alternation of gut microbiota composition.

Fiber source	Treatment	Pig stage	Sampling site	Alteration of microbiota	Health/growth outcomes	Reference
SB	CF + 1.8%, 3.1%, and 4.4%	Duroc × Bamei, averaging 3 months	Jejunum, cecum	↑Bacteroidota and *Turicibacter*	–	(64)
WB	NDF + 150.5 g/kg DM	German Landrace × Piétrain, averaging 3 months	Rectum	↑*Lactobacillus* and bifidobacteria; and ↓*Enterobacteriaceae*	–	(67)
WB	NDF + 1.5%	Large White × Landrace × Pietrain, weaned early	Colon	↓Enterobacteria	↓Mortality; and↓diarrheal rate	(62)
PF and WB	+ xylose 6.92%, arabinose 5.52%; and + glucose 5.89%	Duroc × Landrace × Yorkshire, weaned at 28 ± 2 d	Cecum	↑*Lactobacillus* and bifidobacteria	↓F/G	(25)
SB	+ galactose 1%	Duroc × Landrace × Yorkshire, weaned at 28 ± 2 d	Ileum, cecum	↓*Lactobacillus* (ileum); and ↑*E. coli* (cecum)	↓ADFI	(25)
CB and WB	TDF + 20 g/kg DM	Duroc × Landrace × Yorkshire, weaned at 28 days	Rectum	↑Actinobacteria, Bacillota or *Fibrobacteres*	↑ADG; and↓F/G	(68)
WB	TDF + 2.4%	Suhuai castrated boars	Cecum, colon	↑*Acetitomaculum* and *Butyrivibrio*	↑ADG; and ↓F/G	(61)
PF	NDF + 3%	Duroc × Landrace × Yorkshire, weaned at 28 days	Colon	↑*Lactobacillus*	↑ADFI; and ↓F/G	(49)
Rye and wheat	SDF + 47%; sNSP + 118%	German Landrace, weaned at 28 days	Jejunum, colon, rectum	↑Bacillota; and ↓Pseudomonadota	–	(69)
RSM and SBM	iNSP + 15%; A/X-ratio + 35%	German Landrace, weaned at 28 days	Jejunum, colon, and rectum	↓Bacillota; ↑actinobacteria, Pseudomonadota (jejunum); and ↑Bacteroidota (colon)	–	(69)
PH and OB	IDF + 86 g/kg, 80 g/kg	Duroc × Landrace × Yorkshire, 32.42 ± 1.95 kg	Ileum, colon	↓*Clostridiaceae* (ileum) and *Streptococcus* (colon)	↓BW, ADFI, and ABWG; and ↑intestinal barrier and immune function	(61)
AG	CF + 1.11%	Duroc × Landrace × Large White, aged 35 days	Duodenum, jejunum, and cecum	↑*Paenibacillus* (duodenum); ↑*Paenibacillus*, *Lactococcus*, *Enterococcus*, and ↓*Mycoplasma*; (jejunum); and ↓*Helicobacter* (cecum)	↓Diarrheal rate	(65)
OG	CF + 1.1%	Duroc × Landrace × Large White, aged 35 days	Duodenum, jejunum, and cecum	↑*Paenibacillus* (duodenum); and↓*Helicobacter* (cecum)	↓Diarrheal rate	(65)
PF, WB, and SB	NDF + 8.2%	Duroc × Landrace × Yorkshire, weaned at 28 days	Ileum, colon	↑*Lactobacillus* (ileum); and↑*Bifidobacterium* (colon)	↑Intestinal barrier function	(70)
WB and SB	NDF + 27.4%	Duroc × Landrace × Yorkshire), weaned at 28 days	Ileum	↓*E. coli*	↑Intestinal barrier function	(70)
KF, LC, and CS	SDF + 55%, 60%	Lactating sows—*in vitro*	Rectum	↑*Anaerovibrio*	/	(71)
Xylan	Xylan + 5.59%	Growing barrow, 59.7 ± 2.6 kg	Colon	↑*Bifidobacterium pseudocatenulatum*	Prevent dysbiosis	(63)
Phytolin + fiber	Phytolin + fiber + 1 g	Unknown	Colon	↑*Lactobacillus* and *Catenibacterium*; and↓*Mogibacterium* and *Escherichia–Shigella* complex	–	(72)

ABWG, average body weight gain; ADG, average daily gain; ADFI, average daily feed intake; AG, alfalfa meal; AX, arabinoxylan; A/X ratio, arabinose/xylose; BW, body weight; CB, maize fiber; CL, cellulose; F/G, feed-to-gain ratio; GM, glucomannan; KF, konjac flour; LC, lignocellulose; MCS, modified cassava starch; OB, oat bran; OG, concentrated fiber; PEC, pectin; PF, pea fiber; PH, pea hull; phytolin + fiber, polyphenol-rich sugarcane extract, sugarcane fiber; RSF, rapeseed hulls; RSM, rapeseed meal; SB, soybean; SBM, soybean meal; WB, wheat bran.

**Figure 2:**
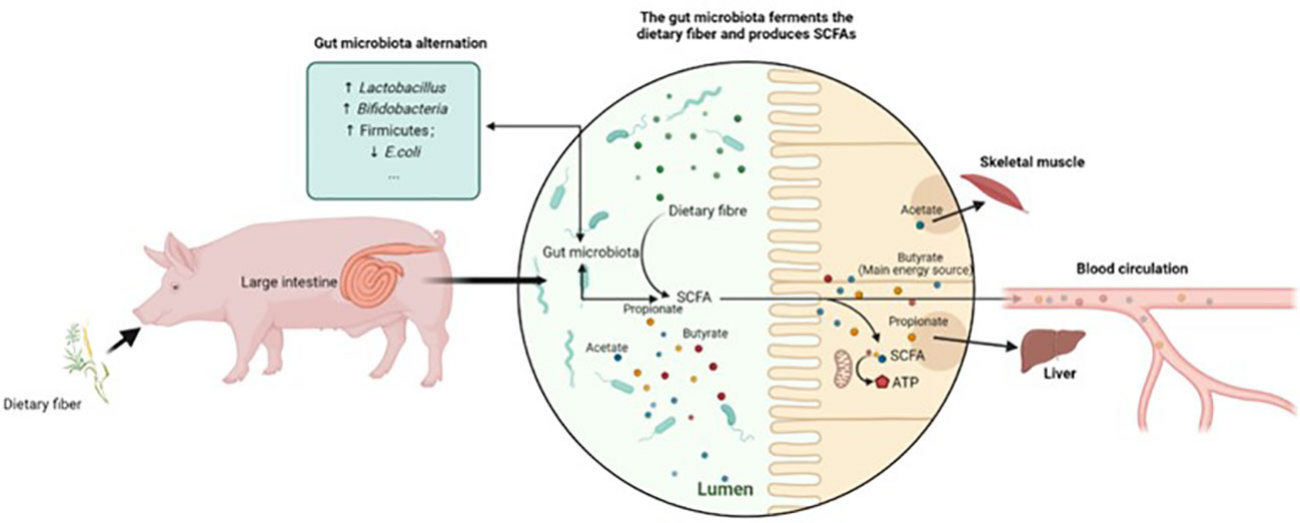
An example of gut microbiota alteration facilitated by the microbial fermentation of DF.

The effect of DF on the alteration of gut microbiota composition is associated with fermentation metabolites, particularly SCFAs (**Figure 2**). Higher abundances of actinobacteria, and Bacillota or *Fibrobacteres*, could promote butyrate production, as Bacillota and actinobacteria are the dominant bacteria that produce butyrate (68). Heinritz et al. observed a positive correlation between acetate and butyrate production and colonic bifidobacteria and *Lactobacillus.* Moreover, decreased enterobacteria in feces and increased production of butyrate in the colon were observed with the addition of wheat bran to pigs’ diets (62). SCFA production influences pigs’ physiological and immune functioning; thus, the variation in individual SCFA concentrations due to distinct degrees of DF fermentation could provide insights into improving pig health and performance. Therefore, the gut microbiota varies depending on the pig life stage and GIT segment, displaying a spatially heterogeneous phenomenon. Bacteria exhibit substrate preference toward specific fiber characteristics, regulating the gut microbiota composition by promoting the growth of bacteria that are more capable of fermenting specific fibers. This resulted in the further variation in SCFA concentrations.

### 4.3 Health-promoted effect of DF via gut microbiota manipulation

The gut microbiota community is composed of a specific ratio of various bacterial species, in which species alternately restrict each other’s function and depend on each other to create an ecological balance. An imbalance in the microbiota community causes gut dysbiosis, which contributes to the development of diseases in pigs, including respiratory infection (73), postweaning diarrhea (74), impairment of the gut–liver axis (75), and intestinal barrier dysfunction (76). The fact that DF can alter microbiota composition indicates that the gut microbiota can be manipulated by DF as a way of improving the health of pigs. It has been reported that insufficient DF intake disturbs the microbiota community, leading to the damage of mucosal layers and increased pathogen susceptibility (77). Wang et al. observed that DF deprivation caused the consistent extinction of *Bifidobacterium* and *Lactobacillus*, and decreased SCFA concentration in pig ileum and feces, whereas xylan supplementation extenuated dysbiosis by selectively promoting the growth of *Bifidobacterium pseudocatenulatum* in the large intestine. Moreover, a positive correlation was observed between SCFA concentration and *B. pseudocatenulatum* abundance (63), indicating that the restoration of dysbiosis is induced by DF deprivation. Onarma et al. have investigated the beneficial effects of a high-fiber rapeseed diet by replacing soybean meal (SBM) with rapeseed meal (RSF). The authors found that RSF favored the growth of beneficial bacteria, including *Lachnospira* and *Coprococcus*, and suppressed the growth of opportunistic pathogenic bacteria, suggesting that RSF has an anti-inflammatory effect and that it reduces the risk of dysbiosis in weaned pigs (78). Therefore, DF can act as a bioactive compound to exert a regulative effect on gut microbiota by attenuating dysbiosis, promoting or depressing specific microbial abundances, and normalizing the gut environment.

### 4.4 A Hierarchical view of fiber specificity related to microbiota accessibility

Fibers with high specificity can be accessed and fermented by only a restricted number of bacteria, resulting in the promotion of specific bacterial growths, regardless of the environmental condition (79). Rogers et al. observed the response of *Bacteroides thetaiotaomicron* in the human gut to 12 carbohydrates to investigate its utilization preference. The results showed that certain carbohydrates were prioritized in the utilization process of *B. thetaiotaomicron* (80). Moreover, the selective accessibility and utilization of bacteria for specific fibers enables bacterial growth. In terms of this fiber specificity, a hierarchical view has been proposed to classify fibers as either low hierarchy or high hierarchy. Low-hierarchy fibers, such as inulin, can be accessed and fermented by a number of bacterial species (79). Thus, competition is present among bacterial species in the fermentation process, depending on their ability to ferment fibers. In the case of high-hierarchy fibers, which generally contain a high insoluble fraction, a limited number of bacteria can access and effectively ferment them. DFs are classified as high hierarchy, mostly because of their complex physicochemical structure, that is, their insoluble matrices and linkage and branch types (81, 82). In general, the more complex the structures are, the fewer bacteria can access and ferment them. This can be explained by the physical property, that is, their degree of insolubility, which can hinder the accessibility and fiber degradation by enzymes (83). Complete saccharification becomes more difficult when many microbial enzymes are required, because of their complex chemical structure. Among high-hierarchy fibers, competition was much less severe, and the promotion of target bacterial growth was more pronounced than that of low-hierarchy fibers (79).

## 5 DF–microbiota interaction and intestinal health

Intestinal health is determined by a combination of factors, including diet supplementation, mucosa integrity, gut microbiota, and the immune system (13). Metabolites derived from microbial fermentation can be considered a result of the interaction between DF supplementation and gut microbiota, and play a critical role in facilitating intestinal health. In this section, we will primarily discuss the effects of SCFAs on intestinal health.

### 5.1 Maintenance of intestinal integrity and barrier function

SCFAs produced by microbial fermentation of specific DFs result in distinct functions on host health. Cellulose present in oat hulls can produce SCFAs to improve nutrient digestibility and intestinal integrity, and modulate gut microbiota (14). SCFAs, particularly butyrate, produced in the hindgut, can meet 60%–70% of the energy requirements for colonic cells and are largely absorbed in weaning and growing pigs (84, 85). The efficient utilization of energy enabled by SCFAs requires the normal function of the intestinal mucosa, largely depending on the colonic cells that exert the main function. Therefore, the function of SCFAs is closely associated with intestinal mucosa integrity. SCFAs can regulate colonic cell proliferation and growth, thus maintain normal absorption and metabolism functions (85). Furthermore, SCFAs, particularly butyrate, are crucial in enhancing intestinal barrier function, which acts as the first line of defense against pathogens (13). Maintaining the intestinal physical barrier is achieved by promoting global cell differentiation and mucin-related gene expression (86, 87), and enhancing mucus excretion and thickness (88).

### 5.2 Enhancement of immune function and prevention of inflammation

The potential mechanism of the anti-inflammatory effect of DF is associated with the gut microbiota and microbiota-derived SCFAs. For instance, it has been reported that DF was involved in addressing inflammatory colonic damage, possibly *via* the activity of acetate, which may play a role in the regulation of neutrophil recruitment, as shown in an experimental model of colitis (89). SCFAs enhance immune function by interacting with immune cells, such as enterocytes, dendritic cells, and helper T cells, consequently affecting adaptive immunity and the inflammatory response (84). In turn, SCFAs facilitate the development of leukocytes and decrease colonic pH, which favor the growth of beneficial bacteria that produce SCFAs (90). Fang et al. found that supplementation with 1 g/kg sodium butyrate in the diet can lower the incidence of diarrhea in pigs and boost their immunity after weaning. This can be explained by the fact that sodium butyrate mitigates weaning stress by increasing the serum IgG concentration and the IgA^+^ cell population in the distal small intestine, and maintains mucosal integrity (91). The mRNA expression levels of tight junction proteins associated with wound healing in the intestine were found to be increased by sodium butyrate supplementation in the diet (92). Furthermore, it was found that SCFAs initiate innate immune responses when exposed to preadipocytes, implying that the presence of SCFAs is beneficial to immune modulation during inflammation. To prevent excessive inflammation, SCFAs promote the differentiation of regulatory T cells that can suppress effector T-cell function and increase IL-10 production (93). Thus, SCFAs could play an essential role in the maintenance of a healthy intestine by regulating immune responses and preventing inflammation.

### 5.3 Immunomodulatory effect in microbial infection

A variety of studies have demonstrated the immunomodulatory role of SCFAs in bacterial infections (94) (95). It was found that acetate could enhance the innate immune response to *Clostridium difficile* by interacting with neutrophils and innate lymphoid cells (96); furthermore, hosts infected with *C. difficile* were detected to have lower levels of butyrate-producing bacteria (97). An increase in butyrate concentrations diminishes *C. difficile* colonization, suggesting that butyrate has an effective role to play in the prevention of bacterial infections (97, 98). The rate of butyrate-producing bacteria in fecal samples was found to decrease under inflammatory bowel disease (IBD) (99). When IBD occurs, intestinal macrophages are largely replaced by monocyte cells circulating in the blood (99, 100). These monocytes eventually achieve maturation in the intestinal lamina propria and obtain bactericidal properties. Butyrate was reported to promote bactericidal properties by stimulating the metabolic shifts of macrophages, and initiating the production of antimicrobial peptides to increase bactericidal activity (100). Studies conducted on human subjects showed that butyrate could act as an anti-inflammatory factor by suppressing nuclear factor kappa B (NF-κB) and interferon gamma (IFN-γ). NF-κB signaling pathways are crucial to the immune response against microbial pathogens, as they are involved in the transcriptional modulation of cytokines, that is, tumor necrosis factor alpha (TNF-α), which actively interacts with the prevention of microbial activity in infections such as *Mycobacterium tuberculosis* (101). Furthermore, feeding fibers is an effective way to enrich the SCFA-producing bacterial population *via* extensive microbial fermentation. Overall, SCFAs have been shown to be indirectly involved in immunomodulation *via* molecular pathways and cellular processes to control and reduce the severity of microbial infection, suggesting the vital role of SCFAs in host−pathogen interactions.

### 5.4 DF, gut microbiota, and intestinal pathology

GIT impairments, including constipation, drooling, dysphagia, and gastroparesis, have been reported in Parkinson’s disease (PD) in humans (102–104). Emerging studies have observed gut microbiota alterations in patients with PD. For instance, *Akkermansia* and *Lactobacillus* were increased (105, 106), and *Prevotella* and *Faecalibacterium* were decreased (105, 106) in PD patients. *Prevotella* and *Faecalibacterium* are SCFA-producing bacteria species; hence, their decreased presence decreases SCFA concentration in PD patients (107). This implies that there is a potential correlation between gut microbiota alteration and intestinal pathology that further impacts SCFA production. Bishehsari et al. found that colon polyposis was associated with gut microbiota dysbiosis, characterized by decreased SCFAs and bacteria, in a rat model. High-fiber supplements have been regarded as an effective treatment, leading to increased SCFA concentrations, and, therefore, a reduction in the severity of symptoms associated with polyposis (108). The protection against colon carcinogenesis could be explained by the fact that DF exhibits a prebiotic effect and favors the growth of beneficial bacteria. Furthermore, increased SCFA production has been reported to modulate cancerous epithelial cells, and exert anti-inflammatory effects in the colon (108). Therefore, existing evidence has revealed the interplay between DF, gut microbiota, and intestinal pathology, and has shown that fermentation metabolites can act as regulatory compounds in the intestinal pathological process.

### 5.5 Maintenance of an anaerobic environment

Microbial fermentation is a process in which the gut environment shifts from being aerobic to anaerobic. SCFAs, particularly butyrate, play key roles in maintaining the gut anaerobic environment and gut homeostasis. During dysbiosis in the gut environment, DF supplementation provides an opportunity for anaerobic bacteria to use fermentative substrates to produce butyrate (13). In homeostatic situations, intestinal tissues utilize butyrate as an energy source through β-oxidation, a process of consuming oxygen, contributing to the maintenance of an anaerobic environment (109, 110). Alternatively, intestinal cells gain energy by anaerobic glycolysis, which can increase the oxygen concentration in the gut environment, resulting in the proliferation of harmful facultative bacteria, such as *Salmonella* (110).

## 6 Concluding remarks

In this review, we addressed recent findings regarding different DFs’ alteration of the gut microbiota profile. The components and physicochemical properties of DF, such as solubility, have been an important factor affecting fiber-degrading bacterial growth, and, consequently, influencing host performance and health. This results in distinct SCFA production, which plays a vital role in influencing intestinal health, since SCFAs maintain normal intestinal function, participate in immune regulation against inflammation and microbial infection, and maintain gut homeostasis. A variety of studies have demonstrated the beneficial effect of DF, namely its promotion of SCFA-producing bacteria, which in turn promotes intestinal health and pig health and performance. This supportive evidence has driven us to gain new insight into proper fiber selection when it is associated with different pig life stages and health statuses to optimize the gut microbiota profile. However, the adverse effects of fibers, such as their anti-nutritional effects, binding toxins, and reduction of nutrient digestibility, should also be taken into consideration. Relevant future research could emphasize DF supply from the perspective of optimizing the gut microbiota profile, thus improving DF feeding strategies in future practice.

## Author contributions

RH conceived and designed the entire review, and wrote the draft. HD, WT, JY, XW, MZ, PH, TW, HF, CZ, CM, YW, and SK reviewed and edited the draft. All authors contributed to the article and approved the submitted version.

## References

[1] Kim HB Isaacson RE. The pig gut microbial diversity: understanding the pig gut microbial ecology through the next generation high throughput sequencing. Vet Microbiol (2015) 177(3–4):242–51. doi: 10.1016/j.vetmic.2015.03.014 25843944

[2] Patil Y Gooneratne R Ju XH. Interactions between host and gut microbiota in domestic pigs: a review. Gut Microbes (2020) 11(3):310–34. doi: 10.1080/19490976.2019.1690363 PMC7524349 31760878

[3] Dominguez–Bello MG Godoy–Vitorino F Knight R Blaser MJ. Role of the microbiome in human development. Gut. (2019) 68(6):1108–14. doi: 10.1136/gutjnl-2018-317503 PMC6580755 30670574

[4] Ursell LK Metcalf JL Parfrey LW Knight R. Defining the human microbiome. Nutr Rev (2012) 70(suppl_1):S38–44. doi: 10.1111/j.1753-4887.2012.00493.x PMC3426293 22861806

[5] Fouhse JM Zijlstra RT Willing BP. The role of gut microbiota in the health and disease of pigs. Anim Frontiers. (2016) 6(3):30–6. doi: 10.2527/af.2016-0031

[6] Brestoff JR Artis D. Commensal bacteria at the interface of host metabolism and the immune system. Nat Immunol (2013) 14(7):676–84. doi: 10.1038/ni.2640 PMC4013146 23778795

[7] Bernad–Roche M Bellés A Grasa L Casanova–Higes A Mainar–Jaime RC. Effects of dietary supplementation with protected sodium butyrate on gut microbiota in growing–finishing pigs. Animals. (2021) 11(7):2137. doi: 10.3390/ani11072137 34359264 PMC8300649

[8] Looft T Allen HK Cantarel BL Levine UY Bayles DO Alt DP. Bacteria, phages and pigs: the effects of in–feed antibiotics on the microbiome at different gut locations. ISME J (2014) 8(8):1566–76. doi: 10.1038/ismej.2014.12 PMC4817603 24522263

[9] Isaacson R Kim HB. The intestinal microbiome of the pig. Anim Health Res Rev (2012) 13(1):100–9. doi: 10.1017/S1466252312000084 22853934

[10] So D Whelan K Rossi M Morrison M Holtmann G Kelly JT. Dietary fiber intervention on gut microbiota composition in healthy adults: a systematic review and meta–analysis. Am J Clin Nutr (2018) 107(6):965–83. doi: 10.1093/ajcn/nqy041 29757343

[11] Cantarel BL Lombard V Henrissat B. Complex carbohydrate utilization by the healthy human microbiome. PLoS One (2012) 7(6):e28742. doi: 10.1371/journal.pone.0028742 22719820 PMC3374616

[12] Jha R Fouhse JM Tiwari UP Li L Willing BP. Dietary fiber and intestinal health of monogastric animals. Front Vet Sci (2019) 6:48. doi: 10.3389/fvets.2019.00048 30886850 PMC6409295

[13] Jha R Berrocoso JD. Dietary fiber utilization and its effects on physiological functions and gut health of swine. Animal. (2015) 9(9):1441–52. doi: 10.1017/S1751731115000919 PMC4574174 25997437

[14] Zhang W Li D Liu L Zang J Duan Q Yang W. The effects of dietary fiber level on nutrient digestibility in growing pigs. J Anim Sci Biotechnol (2013) 4(1):1–7. doi: 10.1186/2049-1891-4-17 23587355 PMC3643821

[15] Lindberg JE. Fiber effects in nutrition and gut health in pigs. J Anim Sci Biotechnol (2014) 5(1):1–7. doi: 10.1186/2049-1891-5-15 24580966 PMC3975931

[16] Tungland BC Meyer D. Nondigestible oligo–and polysaccharides (Dietary fiber): their physiology and role in human health and food. Compr Rev Food Sci Food Saf. (2002) 1(3):90–109. doi: 10.1111/j.1541-4337.2002.tb00009.x 33451232

[17] Tejeda O J Kim W K. Role of dietary fiber in poultry nutrition. Animals. (2021) 11(2):461. doi: 10.3390/ani11020461 33572459 PMC7916228

[18] Dikeman CL Fahey GC Jr. Viscosity as related to dietary fiber: a review. Crit Rev Food Sci Nutr (2006) 46(8):649–63. doi: 10.1080/10408390500511862 17092830

[19] Mudgil D. The interaction between insoluble and soluble fiber. In: Dietary fiber for the prevention of cardiovascular disease. Elsevier (2017). p. 35–59. doi: 10.1016/B978-0-12-805130-6.00003-3

[20] Zijlstra RT Jha R Woodward AD Fouhse J van Kempen T. Starch and fiber properties affect their kinetics of digestion and thereby digestive physiology in pigs. J Anim Sci (2012) 90(suppl_4):49–58. doi: 10.2527/jas.53718 23365281

[21] Wang LF Beltranena E Zijlstra RT. Diet nutrient digestibility and growth performance of weaned pigs fed sugar beet pulp. Anim Feed Sci Technol (2016) 211:145–52. doi: 10.1016/j.anifeedsci.2015.11.005

[22] Shang Q Ma X Liu H Liu S Piao X. Effect of fibre sources on performance, serum parameters, intestinal morphology, digestive enzyme activities and microbiota in weaned pigs. Arch Anim Nutr (2020) 74(2):121–37. doi: 10.1080/1745039X.2019.1684148 31821028

[23] Tiwari UP Mandal RK Neupane KR Mishra B Jha R. Starchy and fibrous feedstuffs differ in their *in vitro* digestibility and fermentation characteristics and differently modulate gut microbiota of swine. J Anim Sci Biotechnol (2022) 13(1):1–11. doi: 10.1186/s40104-022-00699-y 35501888 PMC9063073

[24] Karr–Lilienthal LK Kadzere CT Grieshop CM Fahey GC Jr. Chemical and nutritional properties of soybean carbohydrates as related to nonruminants: A review. Livest Prod Sci (2005) 97(1):1–12. doi: 10.1016/j.livprodsci.2005.01.015

[25] Zhao J Liu P Wu Y Guo P Liu L Ma N. Dietary fiber increases butyrate–producing bacteria and improves the growth performance of weaned piglets. J Agric Food Chem (2018) 66(30):7995–8004. doi: 10.1021/acs.jafc.8b02545 29986139

[26] Mackie A Bajka B Rigby N. Roles for dietary fibre in the upper GI tract: The importance of viscosity. Food Res Int (2016) 88:234–8. doi: 10.1016/j.foodres.2015.11.011

[27] Hung YT Zhu J Shurson GC Urriola PE Saqui–Salces M. Decreased nutrient digestibility due to viscosity is independent of the amount of dietary fibre fed to growing pigs. Br J Nutr (2022) 127(2):177–87. doi: 10.1017/S0007114521000866 PMC8756099 33706826

[28] Hooda S Metzler–Zebeli BU Vasanthan T Zijlstra RT. Effects of viscosity and fermentability of dietary fibre on nutrient digestibility and digesta characteristics in ileal–cannulated grower pigs. Br J Nutr (2011) 106(5):664–74. doi: 10.1017/S0007114511000985 21554809

[29] Bai S Zhang P Lin M Lin W Yang Z Li S. Microbial diversity and structure in the gastrointestinal tracts of two stranded short–finned pilot whales (Globicephala macrorhynchus) and a pygmy sperm whale (Kogia breviceps). Integr Zool (2021) 16(3):324–35. doi: 10.1111/1749-4877.12502 PMC9292824 33174288

[30] Lan G Chen H Chen S Tian J. Chemical composition and physicochemical properties of dietary fiber from polygonatum odoratum as affected by different processing methods. Food Res Int (2012) 49(1):406–10. doi: 10.1016/j.foodres.2012.07.047

[31] Knudsen KEB Lærke HN Jørgensen H. Carbohydrates and carbohydrate utilization in swine. Sustainable swine nutrition (2013). p. 109–37. doi: 10.1002/9781119583998.ch6

[32] Bachmann M Michel S Greef JM Zeyner A. Fermentation characteristics and *in vitro* digestibility of fibers and fiber–rich byproducts used for the feeding of pigs. Animals. (2021) 11(2):341. doi: 10.3390/ani11020341 33572852 PMC7911969

[33] Yang P Zhao J. Variations on gut health and energy metabolism in pigs and humans by intake of different dietary fibers. Food Sci Nutr (2021) 9(8):4639–54. doi: 10.1002/fsn3.2421 PMC8358348 34401110

[34] Chattopadhyay MK. Use of antibiotics as feed additives: a burning question. Front Microbiol (2014) 5:334. doi: 10.3389/fmicb.2014.00334 25071747 PMC4078264

[35] Shang Q Liu H Wu D Mahfuz S Piao X. Source of fiber influences growth, immune responses, gut barrier function and microbiota in weaned piglets fed antibiotic–free diets. Anim Nutr (2021) 7(2):315–25. doi: 10.1016/j.aninu.2020.12.008 PMC8245821 34258419

[36] Kiela PR Ghishan FK. Physiology of intestinal absorption and secretion. Best Pract Res Clin Gastroenterol (2016) 30(2):145–59. doi: 10.1016/j.bpg.2016.02.007 PMC4956471 27086882

[37] Mosenthin R Hambrecht E Sauer WC. Utilisation of different fibres in piglet feeds. recent advances in animal nutrition. Nottingham University Press (1999). pp. 227–56.

[38] Redondo LM Chacana PA Dominguez JE Fernandez Miyakawa ME. Perspectives in the use of tannins as alternative to antimicrobial growth promoter factors in poultry. Front Microbiol (2014) 5:118. doi: 10.3389/fmicb.2014.00118 24723916 PMC3973907

[39] Jha R Leterme P. Feed ingredients differing in fermentable fibre and indigestible protein content affect fermentation metabolites and faecal nitrogen excretion in growing pigs. Animal. (2012) 6(4):603–11. doi: 10.1017/S1751731111001844 22436276

[40] Jha R Berrocoso JFD. Dietary fiber and protein fermentation in the intestine of swine and their interactive effects on gut health and on the environment: A review. Anim Feed Sci Technol (2016) 212:18–26. doi: 10.1016/j.anifeedsci.2015.12.002

[41] Giuberti G Gallo A Moschini M Masoero F. New insight into the role of resistant starch in pig nutrition. Anim Feed Sci Technol (2015) 201:1–13. doi: 10.1016/j.anifeedsci.2015.01.004

[42] Han M Wang C Liu P Li D Li Y Ma X. Dietary fiber gap and host gut microbiota. Protein Pept Lett (2017) 24(5):388–96. doi: 10.2174/0929866524666170220113312 28219317

[43] Maswanganye GMT Liu B Che D Han R. Effects of dietary fiber levels and composition on the intestinal health of finishing pigs. Open J Anim Sci (2021) 11(3):384–98. doi: 10.4236/ojas.2021.113028

[44] Agyekum AK Regassa A Kiarie E Nyachoti CM. Nutrient digestibility, digesta volatile fatty acids, and intestinal bacterial profile in growing pigs fed a distillers dried grains with solubles containing diet supplemented with a multi–enzyme cocktail. Anim Feed Sci Technol (2016) 212:70–80. doi: 10.1016/j.anifeedsci.2015.12.006

[45] Zhang Y Yang L Zhao N Hong Z Cai B Le Q. Soluble polysaccharide derived from laminaria japonica attenuates obesity–related nonalcoholic fatty liver disease associated with gut microbiota regulation. Mar Drugs (2021) 19(12):699. doi: 10.3390/md19120699 34940698 PMC8706399

[46] Hu J Lin S Zheng B Cheung PCK. Short–chain fatty acids in control of energy metabolism. Crit Rev Food Sci Nutr (2018) 58(8):1243–9. doi: 10.1080/10408398.2016.1245650 27786539

[47] le Gall M Serena A Jørgensen H Theil PK Knudsen KEB. The role of whole–wheat grain and wheat and rye ingredients on the digestion and fermentation processes in the gut–a model experiment with pigs. Br J Nutr (2009) 102(11):1590–600. doi: 10.1017/S0007114509990924 19635175

[48] Bai Y Zhou X Li N Zhao J Ye H Zhang S. *In vitro* fermentation characteristics and fiber–degrading enzyme kinetics of cellulose, arabinoxylan, β–glucan and glucomannan by pig fecal microbiota. Microorganisms. (2021) 9(5):1071. doi: 10.3390/microorganisms9051071 34065679 PMC8156825

[49] Ellner C Wessels AG Zentek J. Effects of dietary cereal and protein source on fiber digestibility, composition, and metabolic activity of the intestinal microbiota in weaner piglets. Animals. (2022) 12(1):109. doi: 10.3390/ani12010109 35011215 PMC8749901

[50] Luo Y Ren W Smidt H Wright ADG Yu B Schyns G. Dynamic distribution of gut microbiota in pigs at different growth stages: Composition and contribution. Microbiol Spectr (2022) 10(3):e00688–21. doi: 10.1128/spectrum.00688-21 PMC9241710 35583332

[51] Zhao W Wang Y Liu S Huang J Zhai Z He C. The dynamic distribution of porcine microbiota across different ages and gastrointestinal tract segments. PLoS One (2015) 10(2):e0117441. doi: 10.1371/journal.pone.0117441 25688558 PMC4331431

[52] Petri D Hill JE van Kessel AG. Microbial succession in the gastrointestinal tract (GIT) of the preweaned pig. Livest Sci (2010) 133(1–3):107–9. doi: 10.1016/j.livsci.2010.06.037

[53] Han Y Liu Z Chen T. Role of vaginal microbiota dysbiosis in gynecological diseases and the potential interventions. Front Microbiol (2021) 12:1538. doi: 10.3389/fmicb.2021.643422 PMC8249587 34220737

[54] Mach N Berri M Estellé J Levenez F Lemonnier G Denis C. Early–life establishment of the swine gut microbiome and impact on host phenotypes. Environ Microbiol Rep (2015) 7(3):554–69. doi: 10.1111/1758-2229.12285 25727666

[55] Jha R Rossnagel B Pieper R van Kessel A Leterme P. Barley and oat cultivars with diverse carbohydrate composition alter ileal and total tract nutrient digestibility and fermentation metabolites in weaned piglets. Animal. (2010) 4(5):724–31. doi: 10.1017/S1751731109991510 22444125

[56] Yang H Huang X Fang S Xin W Huang L Chen C. Uncovering the composition of microbial community structure and metagenomics among three gut locations in pigs with distinct fatness. Sci Rep (2016) 6(1):1–11. doi: 10.1038/srep27427 27255518 PMC4891666

[57] Wu G Tang X Fan C Wang L Shen W Ren S. Gastrointestinal tract and dietary fiber driven alterations of gut microbiota and metabolites in durco× bamei crossbred pigs. Front Nutr (2022) 8:806646. doi: 10.3389/fnut.2021.806646 35155525 PMC8836464

[58] Zhao J Bai Y Tao S Zhang G Wang J Liu L. Fiber–rich foods affected gut bacterial community and short–chain fatty acids production in pig model. J Funct Foods. (2019) 57:266–74. doi: 10.1016/j.jff.2019.04.009

[59] Lee JW. Optimization of canola co–product utilization in swine. South Dakota State University (2020). https://openprairie.sdstate.edu/etd/3654.

[60] Luo Y Liu Y Li H Zhao Y Wright ADG Cai J. Differential effect of dietary fibers in intestinal health of growing pigs: Outcomes in the gut microbiota and immune–related indexes. Front Microbiol (2022) 13. doi: 10.3389/fmicb.2022.843045 PMC8902361 35273589

[61] Che L Chen H Yu B He J Zheng P Mao X. Long–term intake of pea fiber affects colonic barrier function, bacterial and transcriptional profile in pig model. Nutr Cancer. (2014) 66(3):388–99. doi: 10.1080/01635581.2014.884229 24611475

[62] Chen H Mao XB Che LQ Yu B He J Yu J. Impact of fiber types on gut microbiota, gut environment and gut function in fattening pigs. Anim Feed Sci Technol (2014) 195:101–11. doi: 10.1016/j.anifeedsci.2014.06.002

[63] Loo YT Howell K Suleria H Zhang P Gu C Ng K. Sugarcane polyphenol and fiber to affect production of short–chain fatty acids and microbiota composition using *in vitro* digestion and pig faecal fermentation model. Food Chem (2022) 385:132665. doi: 10.1016/j.foodchem.2022.132665 35299023

[64] Heinritz SN Weiss E Eklund M Aumiller T Louis S Rings A. Intestinal microbiota and microbial metabolites are changed in a pig model fed a high–fat/low–fiber or a low–fat/high–fiber diet. PLoS One (2016) 11(4):e0154329. doi: 10.1371/journal.pone.0154329 27100182 PMC4839692

[65] Chen H Mao X He J Yu B Huang Z Yu J. Dietary fibre affects intestinal mucosal barrier function and regulates intestinal bacteria in weaning piglets. Br J Nutr (2013) 110(10):1837–48. doi: 10.1017/S0007114513001293 23656640

[66] Hong J Ndou SP Adams S Scaria J Woyengo TA. Canola meal in nursery pig diets: growth performance and gut health. J Anim Sci (2020) 98(11):skaa338. doi: 10.1093/jas/skaa338 33098648 PMC8060915

[67] Gasa FM Ywazaki M de Segura Ugalde AG Hermes RG Gasó JG Hernández JFP. Administration of loperamide and addition of wheat bran to the diets of weaner pigs decrease the incidence of diarrhoea and enhance their gut maturation. Br J Nutr (2010) 103(6):879–85. doi: 10.1017/S0007114509992637 19889242

[68] Pu G Li P Du T Niu Q Fan L Wang H. Adding appropriate fiber in diet increases diversity and metabolic capacity of distal gut microbiota without altering fiber digestibility and growth rate of finishing pig. Front Microbiol (2020) 11:533. doi: 10.3389/fmicb.2020.00533 32328041 PMC7160236

[69] Liu B Wang W Zhu X Sun X Xiao J Li D. Response of gut microbiota to dietary fiber and metabolic interaction with SCFAs in piglets. Front Microbiol (2018) 2344. doi: 10.3389/fmicb.2018.02344 PMC6172335 30323803

[70] Kraler M Schedle K Schwarz C Domig KJ Pichler M Oppeneder A. Fermented and extruded wheat bran in piglet diets: impact on performance, intestinal morphology, microbial metabolites in chyme and blood lipid radicals. Arch Anim Nutr (2015) 69(5):378–98. doi: 10.1080/1745039X.2015.1075671 26305386

[71] Wang Z Bai Y Pi Y Gerrits WJJ de Vries S Shang L. Xylan alleviates dietary fiber deprivation–induced dysbiosis by selectively promoting bifidobacterium pseudocatenulatum in pigs. Microbiome. (2021) 9(1):1–14. doi: 10.1186/s40168-021-01175-x 34802456 PMC8606072

[72] Sencio V Machado MG Trottein F. The lung–gut axis during viral respiratory infections: the impact of gut dysbiosis on secondary disease outcomes. Mucosal Immunol (2021) 14(2):296–304. doi: 10.1038/s41385-020-00361-8 33500564 PMC7835650

[73] Gresse R Chaucheyras–Durand F Fleury MA van de Wiele T Forano E Blanquet–Diot S. Gut microbiota dysbiosis in postweaning piglets: understanding the keys to health. Trends Microbiol (2017) 25(10):851–73. doi: 10.1016/j.tim.2017.05.004 28602521

[74] Ringseis R Eder K. Heat stress in pigs and broilers: role of gut dysbiosis in the impairment of the gut–liver axis and restoration of these effects by probiotics, prebiotics and synbiotics. J Anim Sci Biotechnol (2022) 13(1):1–16. doi: 10.1186/s40104-022-00783-3 36397130 PMC9673442

[75] Zhang Y Gan Y Wang J Feng Z Zhong Z Bao H. Dysbiosis of gut microbiota and intestinal barrier dysfunction in pigs with pulmonary inflammation induced by mycoplasma hyorhinis infection. mSystems. (2022) 7(4):e00282–22. doi: 10.1128/msystems.00282-22 PMC9426446 35699454

[76] Desai MS Seekatz AM Koropatkin NM Kamada N Hickey CA Wolter M. A dietary fiber–deprived gut microbiota degrades the colonic mucus barrier and enhances pathogen susceptibility. Cell. (2016) 167(5):1339–53. doi: 10.1016/j.cell.2016.10.043 PMC5131798 27863247

[77] Onarman Umu ÖC Fauske AK Åkesson CP Pérez de Nanclares M Sørby R Press CM. Gut microbiota profiling in Norwegian weaner pigs reveals potentially beneficial effects of a high–fiber rapeseed diet. PLoS One (2018) 13(12):e0209439. doi: 10.1371/journal.pone.0209439 30571797 PMC6301702

[78] Cantu–Jungles TM Hamaker BR. New view on dietary fiber selection for predictable shifts in gut microbiota. mBio. (2020) 11(1):e02179–19. doi: 10.1128/mBio.02179-19 PMC7029134 32071263

[79] Rogers TE Pudlo NA Koropatkin NM Bell JSK Moya Balasch M Jasker K. Dynamic responses of b acteroides thetaiotaomicron during growth on glycan mixtures. Mol Microbiol (2013) 88(5):876–90. doi: 10.1111/mmi.12228 PMC3700664 23646867

[80] Sheridan PO Martin JC Lawley TD Browne HP Harris HMB Bernalier–Donadille A. Polysaccharide utilization loci and nutritional specialization in a dominant group of butyrate–producing human colonic Bacillota. Microb Genom (2016) 2(2):e000043. doi: 10.1099/mgen.0.000043 28348841 PMC5320581

[81] Martens EC Kelly AG Tauzin AS Brumer H. The devil lies in the details: how variations in polysaccharide fine–structure impact the physiology and evolution of gut microbes. J Mol Biol (2014) 426(23):3851–65. doi: 10.1016/j.jmb.2014.06.022 PMC4252772 25026064

[82] Hamaker BR Tuncil YE. A perspective on the complexity of dietary fiber structures and their potential effect on the gut microbiota. J Mol Biol (2014) 426(23):3838–50. doi: 10.1016/j.jmb.2014.07.028 25088686

[83] Adebowale TO Yao K Oso AO. Major cereal carbohydrates in relation to intestinal health of monogastric animals: A review. Anim Nutr (2019) 5(4):331–9. doi: 10.1016/j.aninu.2019.09.001 PMC6920401 31890909

[84] Liu Y. Fatty acids, inflammation and intestinal health in pigs. J Anim Sci Biotechnol (2015) 6(1):1–9. doi: 10.1186/s40104-015-0040-1 26361542 PMC4564983

[85] Wrzosek L Miquel S Noordine ML Bouet S Chevalier–Curt MJ Robert V. Bacteroides thetaiotaomicron and faecalibacterium prausnitzii influence the production of mucus glycans and the development of goblet cells in the colonic epithelium of a gnotobiotic model rodent. BMC Biol (2013) 11(1):1–13. doi: 10.1186/1741-7007-11-61 23692866 PMC3673873

[86] Vital M Penton CR Wang Q Young VB Antonopoulos DA Sogin ML. A gene–targeted approach to investigate the intestinal butyrate–producing bacterialcommunity. Microbiome. (2013) 1(1):1–14. doi: 10.1186/2049-2618-1-8 24451334 PMC4126176

[87] McRorie. JW Jr McKeown NM. Understanding the physics of functional fibers in the gastrointestinal tract: an evidence–based approach to resolving enduring misconceptions about insoluble and soluble fiber. J Acad Nutr Diet. (2017) 117(2):251–64. doi: 10.1016/j.jand.2016.09.021 27863994

[88] Shen S Prame Kumar K Wen SW Shim R Wanrooy BJ Stanley D. Deficiency of dietary fiber modulates gut microbiota composition, neutrophil recruitment and worsens experimental colitis. Front Immunol (2021) 12:619366. doi: 10.3389/fimmu.2021.619366 33708211 PMC7940676

[89] Akhtar M Chen Y Ma Z Zhang X Shi D Khan JA. Gut microbiota–derived short chain fatty acids are potential mediators in gut inflammation. Anim Nutr (2021) 8:350–60. doi: 10.1016/j.aninu.2021.11.005 PMC9040132 35510031

[90] Fang CL Sun H Wu J Niu HH Feng J. Effects of sodium butyrate on growth performance, haematological and immunological characteristics of weanling piglets. J Anim Physiol Anim Nutr (Berl). (2014) 98(4):680–5. doi: 10.1111/jpn.12122 24024579

[91] Ma X X Fan P Li LS Qiao SY Zhang GL Li DF. Butyrate promotes the recovering of intestinal wound healing through its positive effect on the tight junctions. J Anim Sci (2012) 90(suppl_4):266–8. doi: 10.2527/jas.50965 23365351

[92] Furusawa Y Obata Y Fukuda S Endo TA Nakato G Takahashi D. Commensal microbe–derived butyrate induces the differentiation of colonic regulatory T cells. Nature. (2013) 504(7480):446–50. doi: 10.1038/nature12721 24226770

[93] Luu M Monning H Visekruna A. Exploring the molecular mechanisms underlying the protective effects of microbial SCFAs on intestinal tolerance and food allergy. Front Immunol (2020) 11:1225. doi: 10.3389/fimmu.2020.01225 32612610 PMC7308428

[94] Ranjbar R Vahdati SN Tavakoli S Khodaie R Behboudi H. Immunomodulatory roles of microbiota–derived short–chain fatty acids in bacterial infections. Biomedicine Pharmacotherapy. (2021) 141:111817. doi: 10.1016/j.biopha.2021.111817 34126349

[95] Fachi JL Sécca C Rodrigues PB de Mato FCP di Luccia B Felipe J de S. Acetate coordinates neutrophil and ILC3 responses against c. difficile through FFAR2. J Exp Med (2020) 217(3):e20190489. doi: 10.1084/jem.20190489 PMC7062529 31876919

[96] Fachi JL de Souza Felipe J Pral LP da Silva BK Corrêa RO de Andrade MCP. Butyrate protects mice from clostridium difficile–induced colitis through an HIF–1–dependent mechanism. Cell Rep (2019) 27(3):750–61. doi: 10.1016/j.celrep.2019.03.054 30995474

[97] Antharam VC Li EC Ishmael A Sharma A Mai V Rand KH. Intestinal dysbiosis and depletion of butyrogenic bacteria in clostridium difficile infection and nosocomial diarrhea. J Clin Microbiol (2013) 51(9):2884–92. doi: 10.1128/JCM.00845-13 PMC3754663 23804381

[98] Peloquin JM Goel G Villablanca EJ Xavier RJ. Mechanisms of pediatric inflammatory bowel disease. Annu Rev Immunol (2016) 34:31–64. doi: 10.1146/annurev-immunol-032414-112151 27168239

[99] Schulthess J Pandey S Capitani M Rue–Albrecht KC Arnold I Franchini F. The short chain fatty acid butyrate imprints an antimicrobial program in macrophages. Immunity. (2019) 50(2):432–45. doi: 10.1016/j.immuni.2018.12.018 PMC6382411 30683619

[100] Maji A Misra R Dhakan DB Gupta V Mahato NK Saxena R. Gut microbiome contributes to impairment of immunity in pulmonary tuberculosis patients by alteration of butyrate and propionate producers. Environ Microbiol (2018) 20(1):402–19. doi: 10.1111/1462-2920.14015 29322681

[101] Borghammer P. Is constipation in parkinson’s disease caused by gut or brain pathology? Parkinsonism Relat Disord (2018) 55:6–7. doi: 10.1016/j.parkreldis.2018.08.014 30181087

[102] Fasano A Visanji NP Liu LWC Lang AE Pfeiffer RF. Gastrointestinal dysfunction in parkinson’s disease. Lancet Neurol (2015) 14(6):625–39. doi: 10.1016/S1474-4422(15)00007-1 25987282

[103] Stocchi F Torti M. Constipation in parkinson’s disease. Int Rev Neurobiol (2017) 134:811–26. doi: 10.1016/bs.irn.2017.06.003 28805584

[104] Bedarf JR Hildebrand F Coelho LP Sunagawa S Bahram M Goeser F. Functional implications of microbial and viral gut metagenome changes in early stage l–DOPA–naïve parkinson’s disease patients. Genome Med (2017) 9:39. doi: 10.1186/s13073-017-0428-y 28449715 PMC5408370

[105] Heintz–Buschart A Pandey U Wicke T Sixel–Döring F Janzen A Sittig–Wiegand E. The nasal and gut microbiome in parkinson’s disease and idiopathic rapid eye movement sleep behavior disorder. Movement Disord (2018) 33(1):88–98. doi: 10.1002/mds.27105 28843021 PMC5811909

[106] Unger MM Spiegel J Dillmann KU Grundmann D Philippeit H Bürmann J. Short chain fatty acids and gut microbiota differ between patients with parkinson’s disease and age–matched controls. Parkinsonism Relat Disord (2016) 32:66–72. doi: 10.1016/j.parkreldis.2016.08.019 27591074

[107] Bishehsari F Engen PA Preite NZ Tuncil YE Naqib A Shaikh M. Dietary fiber treatment corrects the composition of gut microbiota, promotes SCFA production, and suppresses colon carcinogenesis. Genes (Basel). (2018) 9(2):102. doi: 10.3390/genes9020102 29462896 PMC5852598

[108] Salvi PS Cowles RA. Butyrate and the intestinal epithelium: modulation of proliferation and inflammation in homeostasis and disease. Cells. (2021) 10(7):1775. doi: 10.3390/cells10071775 34359944 PMC8304699

[109] Wang RX. Microbiota–derived butyrate in the regulation of intestinal homeostasis. University of Colorado Anschutz Medical Campus Strauss Health Sciences Library (2021). doi: 10.25677/tgpz-nw46

[110] Rivera–Chávez F Zhang LF Faber F Lopez CA Byndloss MX Olsan EE. Depletion of butyrate–producing clostridia from the gut microbiota drives an aerobic luminal expansion of salmonella. Cell Host Microbe (2016) 19(4):443–54. doi: 10.1016/j.chom.2016.03.004 PMC4832419 27078066

